# A Nomogram to predict parotid gland overdose in head and neck IMRT

**DOI:** 10.1186/s13014-016-0650-6

**Published:** 2016-06-08

**Authors:** J. Castelli, A. Simon, B. Rigaud, C. Lafond, E. Chajon, J. D. Ospina, P. Haigron, B. Laguerre, A. Ruffier Loubière, K. Benezery, R. de Crevoisier

**Affiliations:** Centre Eugene Marquis, Radiotherapy, de la Bataille Flandre Dunkerque, F-35000 Rennes, France; Rennes University 1, LTSI, Campus de Beaulieu, Rennes, F-35000 France; INSERM, U1099, Campus de Beaulieu, Rennes, F-35000 France; Centre Eugene Marquis, Medical oncology, Rennes, F-35000 France; CHRU de Tours, Radiotherapy, Tours, F-37550 France; Centre Antoine Lacassagne, Radiotherapy, Nice, F-06100 France

**Keywords:** Nomogram, Adaptive radiotherapy, Head and neck, Parotid gland overdose

## Abstract

**Purposes:**

To generate a nomogram to predict parotid gland (PG) overdose and to quantify the dosimetric benefit of weekly replanning based on its findings, in the context of intensity-modulated radiotherapy (IMRT) for locally-advanced head and neck carcinoma (LAHNC).

**Material and methods:**

Twenty LAHNC patients treated with radical IMRT underwent weekly computed tomography (CT) scans during IMRT. The cumulated PG dose was estimated by elastic registration. Early predictors of PG overdose (cumulated minus planned doses) were identified, enabling a nomogram to be generated from a linear regression model. Its performance was evaluated using a leave-one-out method. The benefit of weekly replanning was then estimated for the nomogram-identified PG overdose patients.

**Results:**

Clinical target volume 70 (CTV70) and the mean PG dose calculated from the planning and first weekly CTs were early predictors of PG overdose, enabling a nomogram to be generated. A mean PG overdose of 2.5Gy was calculated for 16 patients, 14 identified by the nomogram. All patients with PG overdoses >1.5Gy were identified. Compared to the cumulated delivered dose, weekly replanning of these 14 targeted patients enabled a 3.3Gy decrease in the mean PG dose.

**Conclusion:**

Based on the planning and first week CTs, our nomogram allowed the identification of all patients with PG overdoses >2.5Gy to be identified, who then benefitted from a final 4Gy decrease in mean PG overdose by means of weekly replanning.

## Introduction

During the course of intensity-modulated radiotherapy (IMRT) for head and neck cancer (HNC), large anatomical variations may result in delivered doses differing from the planned dose [1]. The literature shows that while dose variations in the clinical target volume appear extremely low [2–5], the percentage of patients with estimated PG overdoses ranges widely from 5 to 70 % [1, 5–10]. With the aim of correcting these PG overdoses, an adaptive radiotherapy (ART) strategy involving one or several replannings during treatment has been investigated [1, 2]. These replannings are, however, time-consuming, as a complete delineation can take up to 2.5 h [11–13] and may not be beneficial for all patients. It is therefore crucial to identify patients with PG overdose and evaluate how ART benefits each individual. Ideally, replanning decisions should be based on early and simple anatomical criteria, such as weight loss or decrease in neck diameter, which have been identified as risk factors for over-irradiation [10, 14–17]. However, a clear correlation between these markers and PG overdose has not yet been established [6]. After having identified early predictors of PG overdose, this dosimetric study had two objectives: 1) to generate a nomogram so as to predict PG overdose; 2) to quantify the benefits of weekly replanning, triggered by the nomogram, in terms of dose sparing and decrease in xerostomia risk.

## Materials and methods

### Patients and tumors

A total of 20 patients (mean age: 63; range: 50–77) were enrolled in this study. All patients presented with locally-advanced oropharyngeal cancer (Stage III or IV, American Joint Committee on Cancer 7th ed.). Patient, tumor, and treatment characteristics have been provided in Table 1.

**Table 1:** Patient, tumor, and treatment characteristics at initial planning (CT0)

ID	Gender	Age	TNM	Tumor sublocation	Chemotherapy	Volume (cm^3^)			Mean planned PG dose (Gy)		Xerostomia NTCP (%) [23, 24]	
						CTV70	ILP	CLP	ILP	CLP	ILP	CLP
1	M	77	T4N0	Tonsil	Cetuximab	45.2	52.1	48.6	30.2	31.1	27.1	28.9
2	F	61	T2N2	Base of tongue	CDDP	26.3	31.1	27.5	31.4	26	29.7	19.1
3	M	70	T3N2c	Oropharynx	CDDP	181.5	24.9	20.7	37.9	31.1	45.1	29.1
4	F	66	T2N2c	Oropharynx	Cetuximab	107.2	27.8	23.4	32.9	27.9	33.1	22.5
5	M	57	T3N0	Velum	CDDP	62.4	20.7	18	28.1	27.8	23	22.3
6	M	67	T3N2c	Base of tongue	CDDP	156.2	24.5	22.7	30.8	29.4	25.4	22.1
7	M	52	T4N2a	Tonsil	Cetuximab	165.1	N/A	21.6	N/A	28.7	N/A	24
8	M	67	T4N1	Base of tongue	CDDP	139.3	22	19.3	30.7	29.2	28	25
9	F	65	T3N3	Base of tongue	CDDP	237.5	23.9	20.2	42.4	31.1	56.1	29
10	F	65	T4N3	Oropharynx	CDDP	257.9	N/A	24.5	N/A	35.2	N/A	38.5
11	M	50	T4N2c	Oropharynx	CDDP	434.5	N/A	17.7	N/A	36.3	N/A	41.1
12	M	53	T3N0	Base of tongue	CDDP	14.4	16.6	23.3	41.3	24.2	53.6	16.3
13	M	73	T3N2c	Oropharynx	Cetuximab	147	29.4	29.2	54.6	32.2	82.1	31.4
14	M	56	T3N0	Epiglottic	Cetuximab	14	22.8	29.2	19.7	9.2	10.3	2.7
15	M	75	T2N2a	Oropharynx	Cetuximab	76.3	20.3	22.4	29.4	29.1	25.6	25
16	M	57	T3N0	Oropharynx	CDDP	46.5	23.8	31.2	32.1	31.2	29.3	31.3
17	M	64	T3N2c	Epiglottic	CDDP	109.8	23.5	15.6	39.6	17.3	49.3	7.8
18	M	55	T1N2b	Tonsil	CDDP	31	20.2	20.8	25.7	23.63	18.7	15.3
19	M	65	T4N0	Velum	CDDP	10.1	23.7	25.3	28.6	28.2	24	23.2
20	M	56	T4N2b	Pharyngeal wall	CDDP	150	32.4	26.8	45	24.4	62.8	16.6

*M* male, *F* female, *CTV70* clinical target volume receiving 70Gy, *ILP* ipsilateral parotid glands, *CLP* contralateral parotid glands, *CDDP* cisplatin, *NTCP* normal tissue complication, *PG* parotid gland, *N/A* not applicable (PGs included in the CTV)

The NTCP Lyman Kutcher Burman (LKB) model (*n =* 1, m = 0.4, and median toxic dose [TD_50_] = 39.9) [23, 24] defined the risk of xerostomia as a salivary flow ratio <25 % of the pretreatment one

### Treatment and planning

All patients underwent IMRT with total doses of 70Gy (2Gy/fraction/day, 35 fractions), combined with a simultaneous integrated boost technique [18] and concomitant chemotherapy (cetuximab or platinum). Planning CTs (CT0) were performed with intravenous contrast agents using 2-mm slice thickness, from the vertex to the carina. Three target volumes were generated: CTV_70,_ CTV_63_, and CTV_56_. The 70Gy clinical target volume (CTV_70_) was equal to the gross tumor volume plus a 5-mm 3D margin, adjusted to exclude the air cavities and all bone mass free of tumor invasion. CTV_63_ corresponded to the area at high-risk of microscopic spread, in particular the ipsilateral nodal level II, while CTV_56_ corresponded to the low-risk subclinical area. The planning target volume (PTV) was the CTVs plus a 5-mm 3D margin, limited at 3 mm from the skin surface in order to avoid the build-up region and therefore limit skin toxicity [19]. The minimum PTV covered by the 95 % isodose line was 95 %. Dose constraints were set according to the GORTEC group (the French group of radiation oncology for head and neck cancer) (Table 2).

**Table 2:** Dose constraints according to the GORTEC group (the French group of radiation oncology for head and neck cancer). D2%: Near maximum absorbed dose

Organ at risk	Dose constraint
Spinal cord	D2% < 45Gy
Brainstem	D2% < 54Gy
Optic nerves	D2% < 54Gy
Contralateral parotid	Mean dose < 30Gy, median dose < 26Gy
Ipsilateral parotid	Mean dose: as low as possible
Oral cavity	Mean dose < 30Gy, V30 < 65 %, and V35 < 35 %
Lips	D2% < 30Gy, mean dose < 20Gy

Parotid sparing was not conducted if considered to the detriment of PTV coverage or other essential organs at risk (OARs).

During the treatment course, set-up errors >5 mm were corrected by weekly in-room stereoscopic kV imaging. Informed consent was obtained from all patients. This study was approved by the institutional review board (ARTIX study NCT01874587).

### Weekly dose estimations

Each patient underwent six weekly CTs (CT1-CT6) using the same protocol as CT0 over the treatment course, except for some variations in intravenous contrast agent use, which was not systematically employed, particularly not in the context of cisplatin-based chemotherapy. Anatomical structures were manually segmented on each weekly CT by the same radiation oncologist for each patient. In the event of complete response, the original macroscopically-involved areas were still included in CTV_70_, which was adjusted to exclude any air cavities and bone mass showing no evidence of original tumor invasion.

**Fig. 1:**
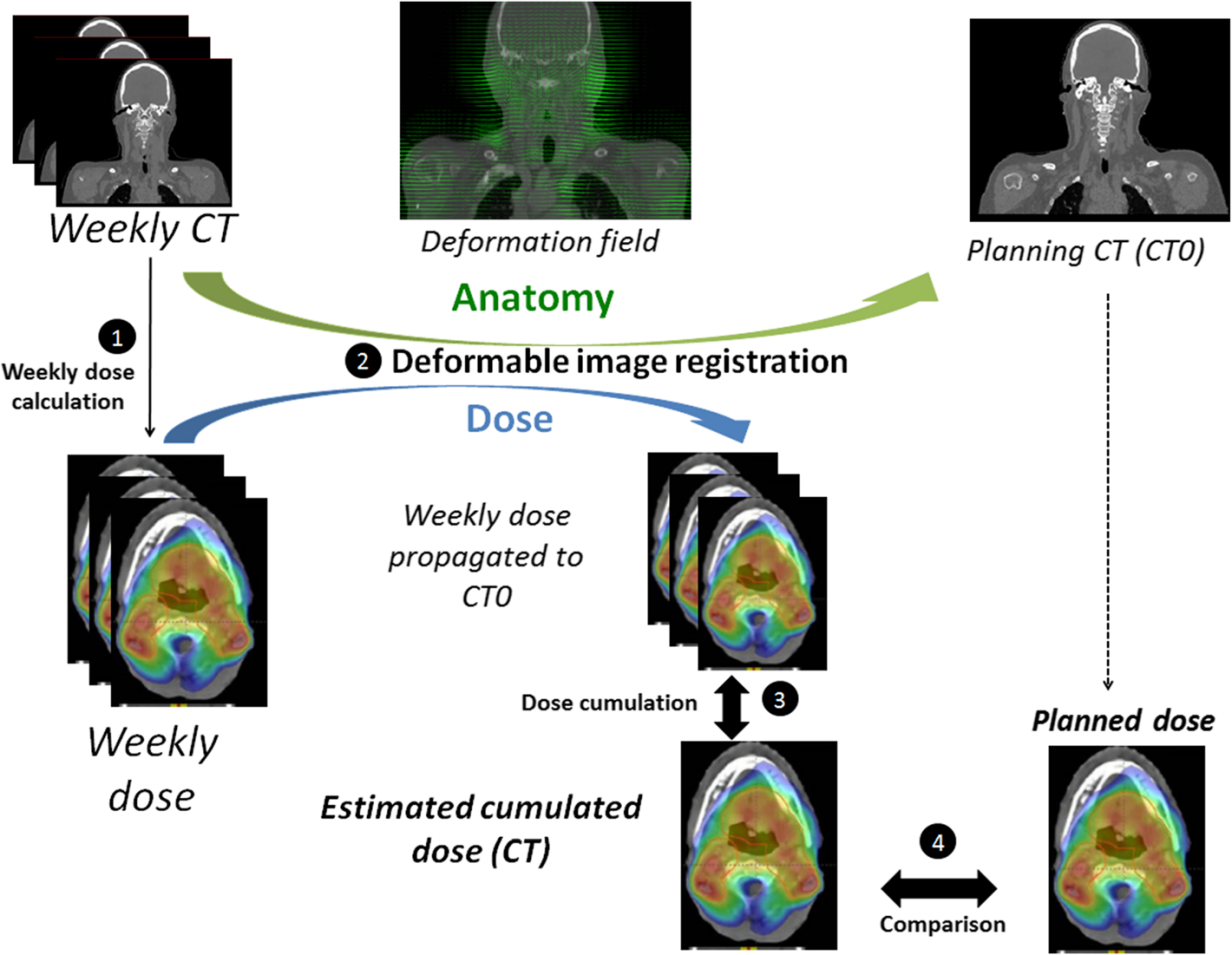
*Total cumulated dose estimation by deformable image registration in four steps.* Step 1: The weekly delivered doses were calculated from the weekly computed tomography scans (CTs). Step 2: A deformable image registration was applied to the weekly doses according to the deformation field between the weekly and planning CT (CT0). Step 3: The propagated dose distributions were totaled to compute the cumulated dose for the CT0. Step 4: The planned dose was compared with the estimated cumulated dose

Treatment always commenced on Mondays, with each weekly CT performed the following Monday. As patients were treated 5 days per week, each weekly CT corresponded to a 10Gy additional dose to the PTV (CT1 at 10Gy, CT2 at 20Gy, and so on). The actual doses delivered weekly were estimated by calculating the dose distribution on the weekly CTs using treatment parameters and the CT0 isocenter (Fig. 1).

### Total cumulated dose estimations using deformable registration

For all patients, the weekly CT images were first registered to the planning CT using a rigid transformation defined by six parameters (three translations and three rotations). The mean squared error was used as a similarity criterion. The cumulated dose estimate relied on deformable image registration, using the free-form deformation (FFD) method. The control points were iteratively displaced according to the considered metric. The dense deformation field was obtained by B-spline interpolation [20]. The mutual information metric was used to handle the modified intensities between CT datasets caused by the presence of contrast agent. The geometric transformation obtained using both rigid and deformable registration was then applied to the weekly dose distributions in order to propagate each one to the planning CT0 dataset. The implementation was provided by the ElastiX library [21].

The average Dice score for PG registration was 0.81 (0.62–0.94). The propagated dose distributions were totaled to compute the cumulated dose on the planning CT, which was finally compared to the planned dose.

### Linear regression model and nomogram to predict PG overdose

PG overdose was calculated as the difference between the cumulated mean PG dose and the mean PG dose of the planning CT dataset (CT0).

**Fig. 2:**
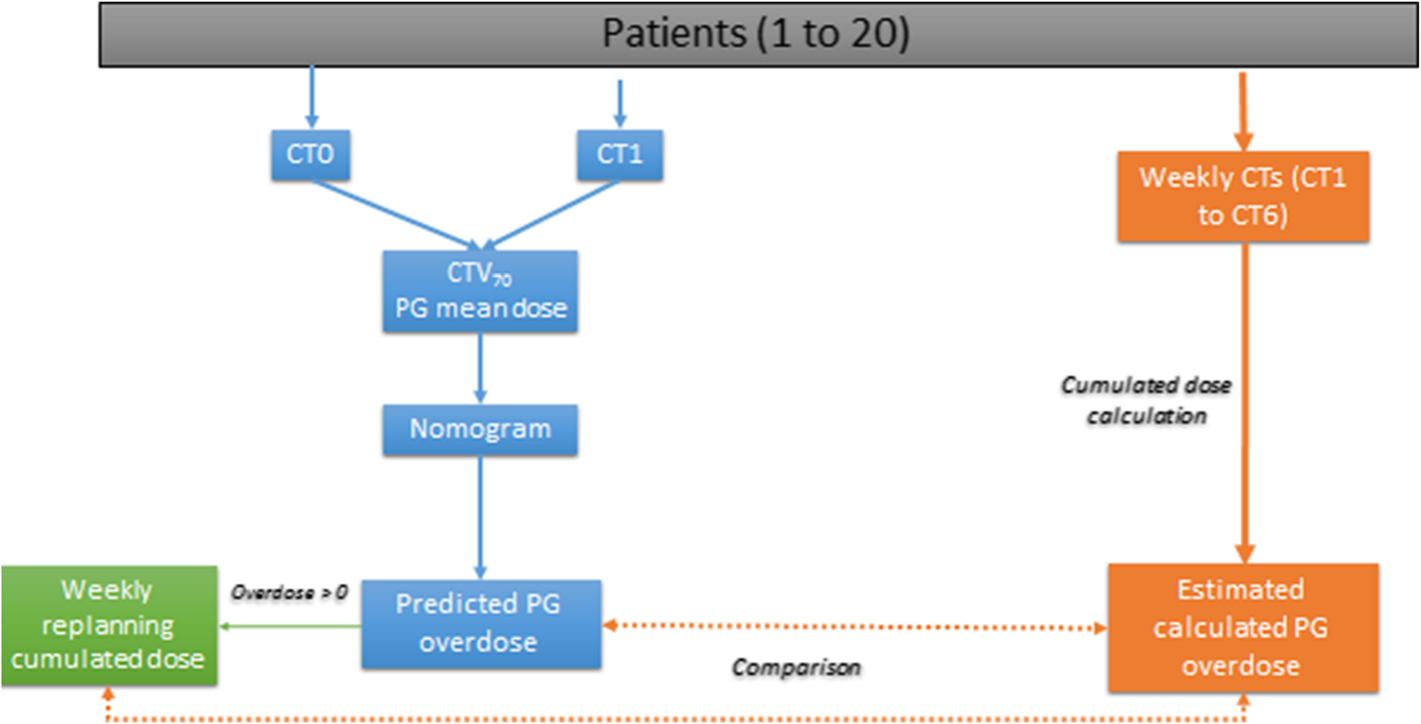
*Nomogram use to predict parotid gland overdose.* For each patient, the nomogram was used to predict the parotid gland (PG) overdose. In the event of an estimated PG overdose, weekly replanning was performed. The cumulated doses with replanning were compared to those without to quantify the benefit of adaptive radiotherapy (ART). CT: computed tomography; CT0: planning CT; CT: first weekly CT (C1); CTV70: clinical target volume receiving 70Gy

The following anatomical and dosimetric parameters have previously been described in the literature as correlating with PG overdose [6, 10, 14, 15], and were assessed for this correlation: CTV70 (cm3), PG volume (cm3), neck thickness (mm), PG-to-CTV70 distance, and mean PG dose. The PG-to-CTV70 distance was defined as the minimal Euclidean distance between the surfaces of the two contours. The difference between and ratio of each of these parameters computed from the two CTs (CT0 and each weekly CT, respectively) were also studied. The Pearson correlation was used to assess the correlation between variables significantly correlated with the PG overdose. When high correlation was observed between two variables (r^2^ > 0.5), only the most significant parameter was included for further analysis. Finally, a linear regression method with backward elimination (coefficient of determination r^2^ > 0.3, *p <*0.05) was used to generate a model for PG overdose prediction. Regression was run with and without an intercept. The standard errors were compared to decide whether ordinary least squares or regression through origin provides a superior fit [22]. The model’s accuracy was then validated by the quantiles-quantiles plot (QQ-plot) and r^2^ for PG overdose prediction. A nomogram was generated based on this model, *i.e.* a chart representing a linear function calculating a predicted value from plotted input data (Fig. 2).

Leave-one-out cross validation was then performed to estimate the model’s stability and accuracy. This method consisted in all patients but one (*n =* 19) being used to develop a PG overdose model (difference between cumulated PG dose and planned dose). A PG overdose prediction was then calculated for the one remaining patient using the model. This predicted PG overdose was then compared to the cumulated PG overdose. This step was repeated for each patient. The variance of each model parameters was calculated to estimate the model’s stability and identify outliers. The mean squared error of the predicted values was calculated.

Statistical analysis was carried out using the Statistical Package for the Social Sciences V. 20.0, and R language and environment for statistical computing.

### Weekly replanning for the patients at risk of PG overdose

Using the nomogram, we identified patients predicted to receive at least one PG overdose, for whom we performed a weekly IMRT replanning on each weekly CT dataset, in accordance with the dose constraints described for the initial planning. PTV coverage did not significantly differ between the initial planning and the weekly replanning. The dose constraints specified for the OARs complied with the GORTEC recommendations for all replanning, as for the initial planning. The mean PG cumulated doses with replanning were compared to those without. The impact of the replanning on the risk of xerostomia was estimated by using the Lyman Kutcher Burman (LKB) model of normal tissue complication probability (NTCP) (*n =* 1, m = 0.4, and median toxic dose [TD_50_] = 39.9) [23, 24], the complication defined as a salivary flow ratio <25 % of the pretreatment one at 12 months.

## Results

A total of 37 PGs were analyzed, due to three ipsilateral PGs included within the PTV being excluded from analysis.

**Fig. 3:**
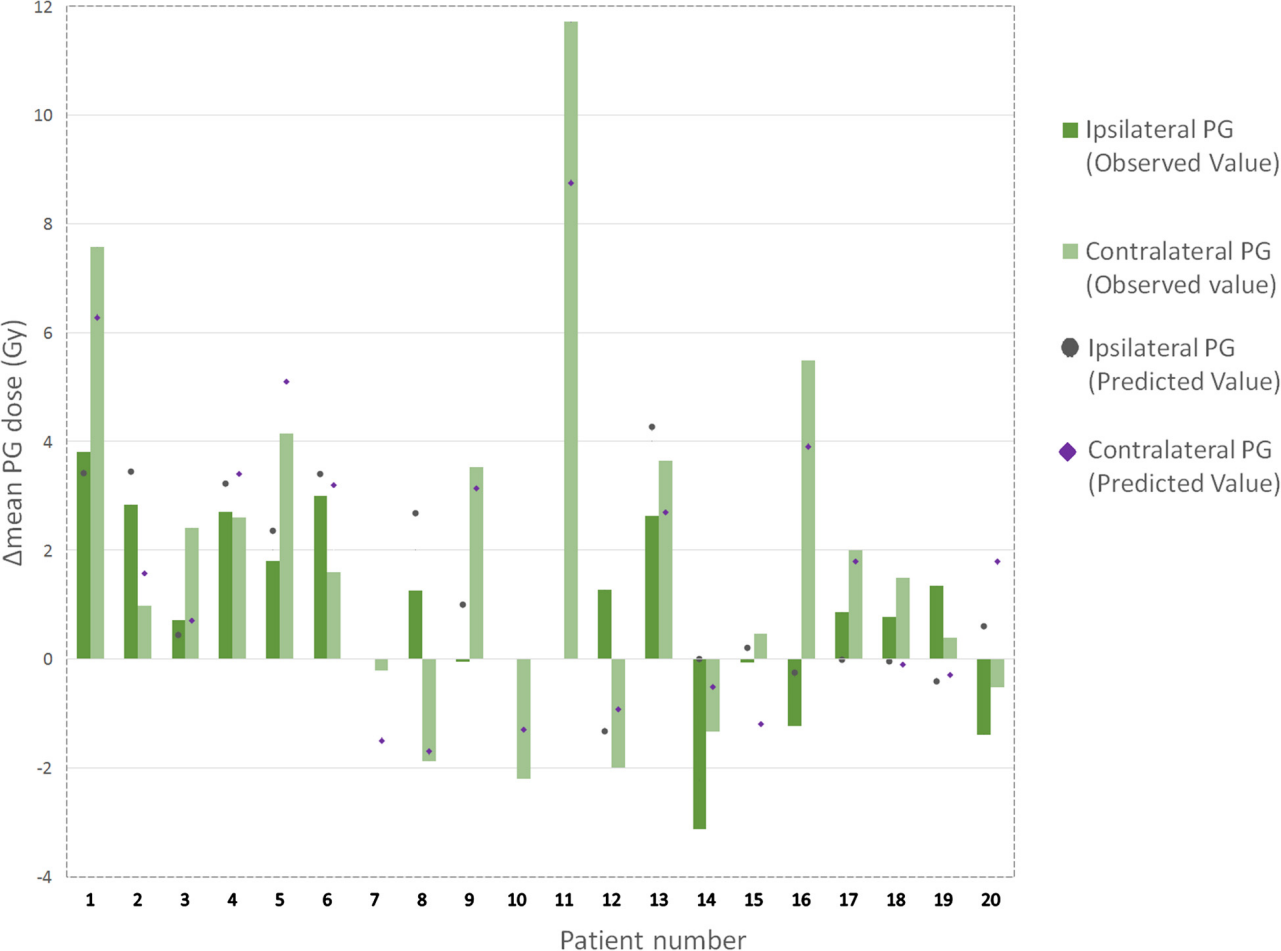
*Parotid gland overdose assessment.* The mean dose difference was calculated between the estimated cumulated dose (without replanning) and the planned dose, in each parotid gland (PG) (ipsilateral and contralateral), for each of the 20 patients. A dose difference with a positive or negative value corresponded to a PG overdose or under-dose, respectively. Predicted PG overdose calculated by the nomogram are represented by circles (ipsilateral PG) or diamonds (contralateral PG) (cf. Section 3.2)

Based on the difference between the cumulated and planned PG doses, two PG subgroups were identified (Fig. 3):

- a PG overdose group: 70 % of all the PGs, with a mean dose increase of 2.5Gy (up to 11.7Gy) and 16 patients presenting at least one overdosed PG;
- a PG under-dose group: involving the other 30 % of the PGs, with a mean dose decrease of 1.2Gy (up to 3.1Gy).

When identifying PG overdose predictors, we found the parameters from the first weekly CTs to be the most significant. Two anatomical and three dosimetric parameters were significantly correlated with PG overdose (Table 3).

**Table 3:** Correlation between the anatomical/dosimetric parameters (calculated on CT0 and CT1) and the PG overdose

Analyzed parameters	r^2^	p-value
CTV_70_CT0_	0.32	0.038
∆CTV_70_	−0.46	0.004
∆DosePG	0.72	<0.001
DmeanPG__CT1_	0.49	0.002
DmeanPG__CT1/_DmeanPG__CT0_	0.70	<0.001

r^2^ = Pearson correlation value, DmeanPG = Mean PG dose (Gy), _CT0 = on the planning CT (CT0), _CT1 = CT at the first week (CT1), ∆ = difference of the parameter between CT1 and CT0

As the mean PG dose for CT1 and the CT1/planning mean PG dose ratio were highly correlated with the difference between the mean PG doses for CT1 and CT0 (∆DosePG) (r^2^ = 0.5 and 0.9, respectively, *p <*0.001), only the parameter with the highest r^2^ was used (mean PG dose difference). The three parameters used for PG overdose prediction were CTV_70_ on the planning CT dataset (CTV_70_CT0_), the CTV_70_ CT0-to-CT1 difference (∆CTV_70_), and the difference between the mean PG doses of CT1 and CT0 (∆DosePG).

Based on these parameters, the resulting linear regression model for PG overdose prediction, optimized for all patients, was:

$$ \mathrm{PGoverdose}=\left(0.007\times \mathrm{C}\mathrm{T}\mathrm{V}7{0}_{\mathrm{CTO}}\right)-\left(0.045\times \Delta \mathrm{CT}\mathrm{V}70\right)+\left(0.509\times \Delta \mathrm{DosePG}\right) $$

**Fig. 4:**
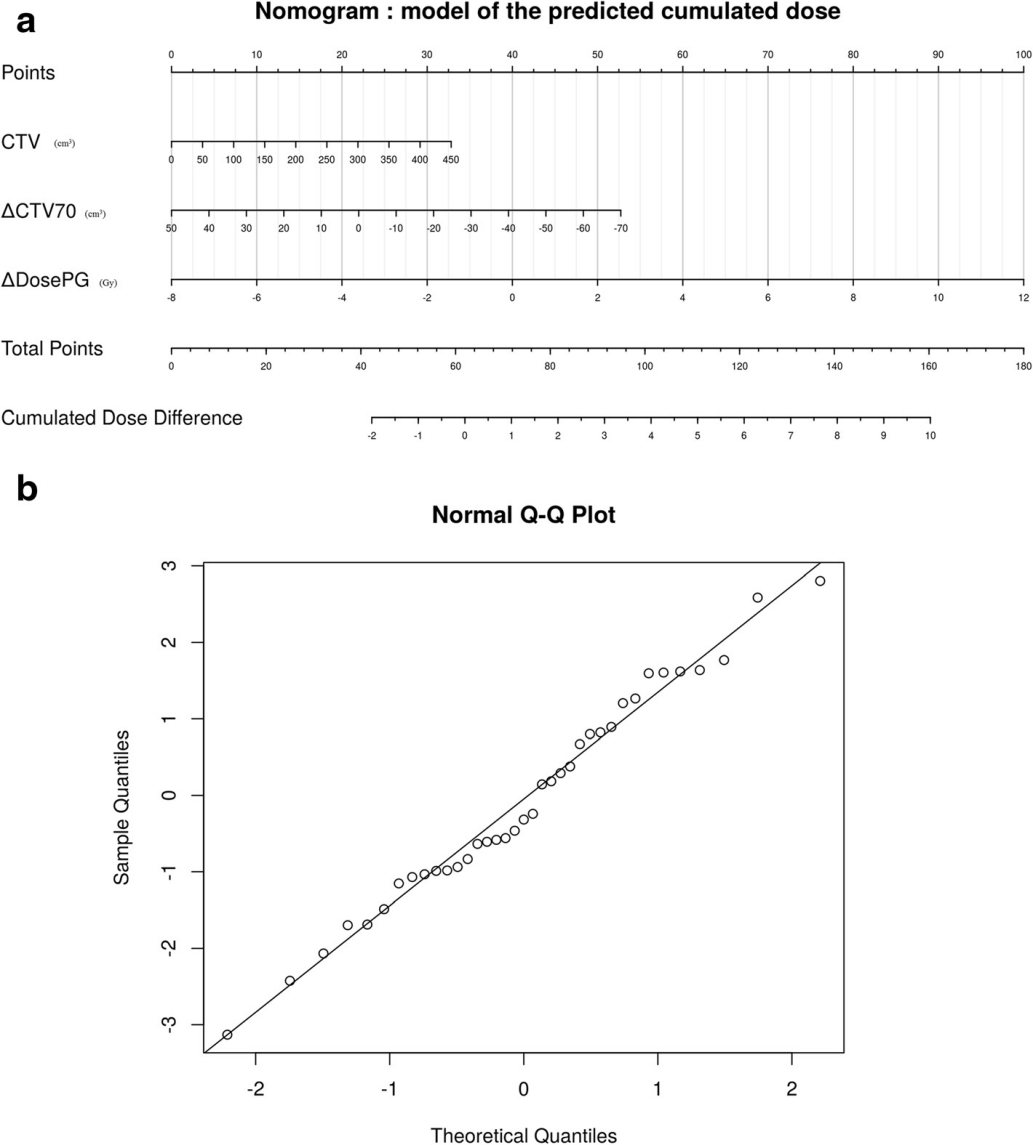
**a** Nomogram to predict PG overdose. The nomogram enables easy prediction of the difference between the mean cumulated parotid gland (PG) dose and the mean planned PG dose (in Gy), using three parameters calculated on the planning computed tomography (CT) (CT0) and on a CT performed during the first week of the treatment (CT1). CTV70 (in cm^3^): clinical target volume receiving 70Gy; ΔCTV70 (in cm^3^): difference between the CTV70 of CT1 and CT0; ΔDosePG (in Gy): mean PG dose difference between CT1 and CT0. **b** Quantiles-quantiles plot (Q-Q plot) of the nomogram. The more accurately the quantile position is aligned, the more linear the model

The corresponding nomogram is shown in Fig. 4a. The quantiles-quantiles plot of the nomogram is shown in Fig. 4b.

**Fig. 5:**
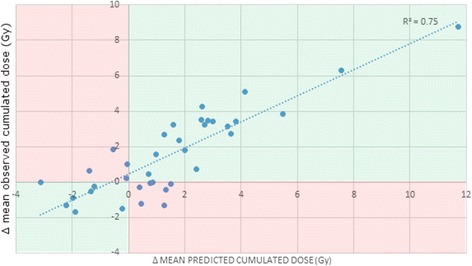
*Correlation between observed and predicted parotid gland (PG) doses.* Representation of the observed (vertical axis) and predicted (horizontal axis) values of PG dose variation (difference between cumulated and planned PG dose) for each PG. The model included three parameters (CTV_70_ shrinkage at the CT1, mean PG dose difference between the dose delivered at CT1 and the planned dose, and CTV_70_ at the planning). Blue line: regression line (R^2^ = 0.75). Red areas: wrong predictions (*e.g.* predicted overdose *vs.* observed under-dose). CT: computed tomography; CTV70 (in cm^3^): clinical target volume receiving 70Gy; CT1: Week 1 treatment CT

The correlation between the observed and predicted cumulated PG doses is shown in Fig. 5 (r^2^ = 0.75).

Dose variations (PG over- or under-doses) were correctly predicted for 25 of the 37 PGs (Fig. 3). Of the 16 patients with at least one overdosed PG, 13 were accurately identified (One patient with an error concerning the side of the PG overdose). In the three patients (Patients 12, 18 and 19) who were inaccurately classified, the mean PG was increased by an average of 1Gy (range: 0.4–1.5Gy).

The model’s performance, evaluated using the leave-one-out cross validation, in terms of identifying patients with or without PG overdose achieved sensitivity, specificity, and positive and negative predictive values of 80 %, 60 %, 86 %, and 50 %, respectively. The mean values and standard deviations (SDs) of each coefficient, considering the leave-one-out cross validation, were 0.007 (SD: 0.0006), −0.045 (SD: 0.004), and 0.507 (SD: 0.01), for $\mathrm{C}\mathrm{T}\mathrm{V}{70}_{\mathrm{CT}0}$_,_$\Delta CTV70$_,_ and $\Delta DosePG$, respectively. The mean square error for the predicted PG overdose was 2.6 Gy (SD: 1.6 Gy). No significant outliers were extracted from the validation procedure.

**Fig. 6:**
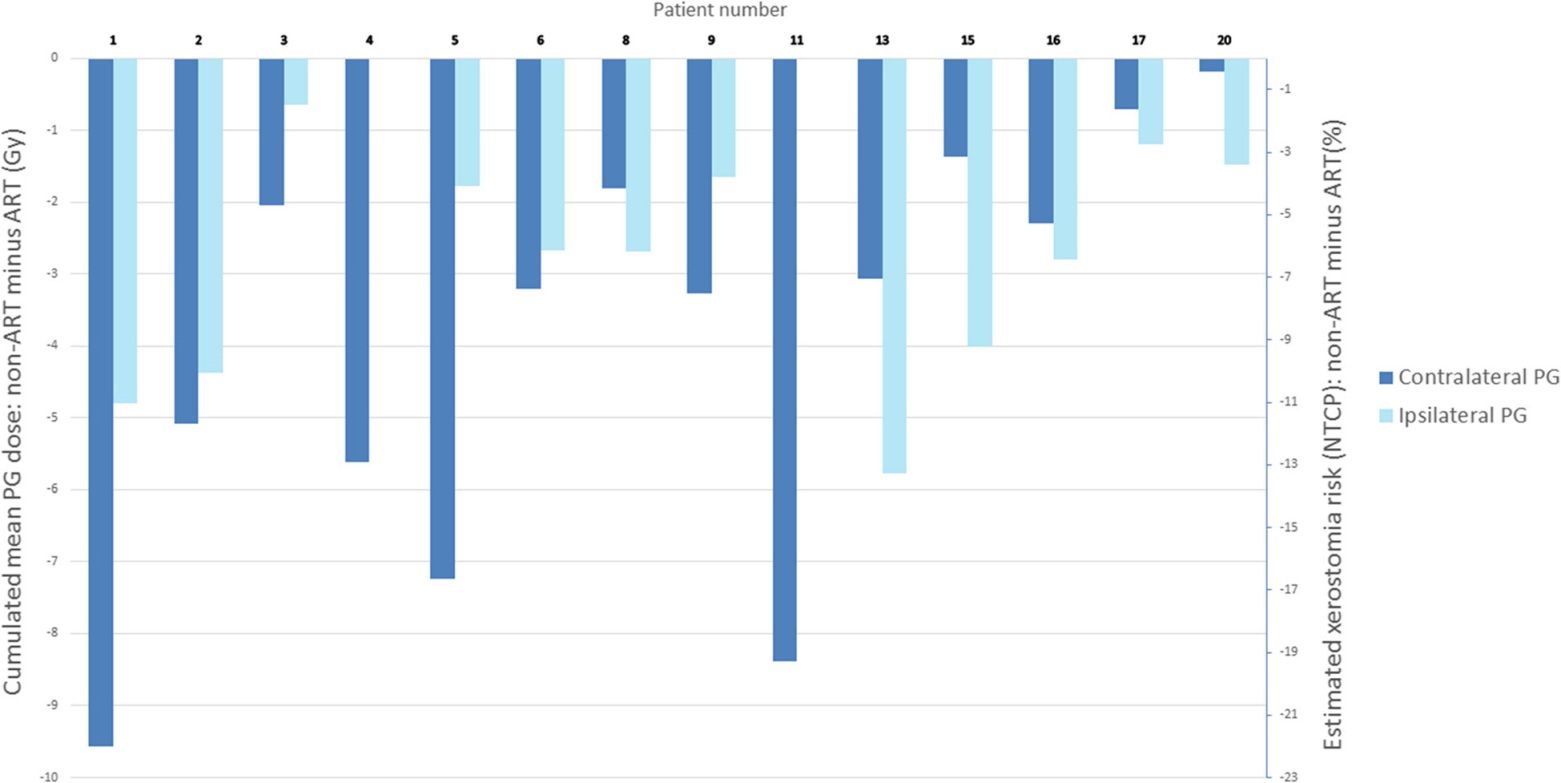
Benefit of weekly replanning for the 14 parotid gland (PG) overdose patients identified by the nomogram. Cumulated mean dose difference between doses with replanning and those without (left y-axis), in each of the parotid glands (PGs) (ipsilateral and contralateral). The corresponding estimated risk of xerostomia (%), computed using the normal tissue complication (NTCP) Lyman Kutcher Burman (LKB) model (LKB NTCP) (n = 1, m = 0.4, and median toxic dose [TD_50_] = 39.9) [23, 24] is represented on the right y-axis. Xerostomia was defined as a salivary flow ratio <25 % of the pretreatment one

For the 14 patients identified by the nomogram as having a predicted PG overdose, the mean PG dose without ART was 34.8Gy (range: 20.9–51.4Gy), corresponding to a mean xerostomia risk of 37 % (20–86 %). The dosimetric benefit of weekly replanning for these 14 patients is shown in Fig. 6. Replanning achieved an average decrease of 3.9Gy in the mean PG dose (range: 0–9.5Gy), representing an average decrease in absolute xerostomia risk of 8 % (0–22 %).

## Discussion

We generated a nomogram aimed at predicting PG overdose based on early predictors calculated on the planning CT dataset and on a CT performed in the first week of treatment.

In total, 14 of the 16 patients with a calculated mean PG overdose of 2.5Gy were identified by the nomogram. All patients with a PG overdose >2.5Gy were identified. Weekly replanning of these 14 targeted patients enabled the mean PG dose to be decreased by 3.9Gy compared to the cumulated delivered dose, corresponding to an 8 % decrease in the estimated absolute xerostomia risk.

Due to anatomical variations occurring during the course of IMRT, some PGs can receive doses exceeding the planned dose (overdose). In an attempt to prevent this, ART is designed to take these anatomical variations into account by generating one or several new plannings. An increasing number of studies demonstrate this technique to have dosimetric benefits [1, 2, 4, 25]. The clinical impact of replanning has been evaluated in two studies [5, 26], where it was shown to improve both patient quality of life [26] and localize disease control, yet had no impact on overall survival. Nevertheless, ART is particularly complex and time-consuming [11–13], generating increased workload for all treatment staff. As not all patients may benefit from ART, it is essential to identify early predictors of PG overdose to enable appropriate patient selection. Neck thickness, weight loss, PG volume, initial tumor volume, and decrease in tumor volume were found to correlate with PG overdose [10, 14, 15]. In a recent review [6], these anatomical parameters were identified as selection criteria for ART patient selection, though no clear conclusion was reached due to the heterogeneity of the studies. However, these correlations were mostly primarily identified using parameters calculated at the end of treatment, therefore significantly limiting the possibility of treatment modifications. These parameters were also correlated with each other. Decrease in PG volume, for instance, has been found to correlate with age, body mass index, planned dose to the parotid glands, initial PG volume, and the volume of PG overlapping with lymph node metastases. Decrease in PG volume may be a useful parameter for identifying patients at higher risk of xerostomia [27]. However, neither the Brouwer et al. [6] study nor our own found any clear association between the decrease in PG volume and PG overdose. Other parameters may be indirectly linked to PG overdose. Human papillomavirus (HPV)-positive cancer has demonstrated a higher sensitivity to radiation [28, 29]. As the decrease in the CTV during the first week of treatment was correlated with PG overdose, HPV-status may exert an impact on the risk of PG overdose. Yet our series was not large enough to analyze the impact of this relationship.

Anatomical variations occurring during the first two weeks of treatment may be particularly relevant for PG overdose prediction, and may justify early replanning, resulting in significant PG dose sparing [17, 30]. Indeed, in the literature, CTV shrinkage appears to be particularly significant during the first week of radiotherapy [31]. In our study, the only anatomical parameters strongly correlated with PG overdose were CTV_70_ at planning and its decrease in the first week. However, this parameter alone was not sufficient to predict final PG overdose. Indeed, the most relevant parameter in our nomogram was the PG dose difference between the planning CT (CT0) and the first week of treatment CT (CT1). Early PG overdose has also been demonstrated to correlate with the estimated cumulated PG dose by Hunter et al. [8].

In our study, we found that considering the parameters only for the planning CT was not sufficient for PG overdose prediction. The acquisition of a new CT, performed during the first week of treatment with dose calculation, was required to predict the mean cumulated PG dose at the end of treatment. In terms of practical use of ART, replanning decisions can be based solely on anatomical parameters, ideally defined on cone-beam CT (CBCT) performed at the time of the fraction, which is also useful for bone registration to correct for patient set-up. This approach should be explored further, assuming that anatomical parameters alone could be sufficient for PG overdose prediction, and also be visible on the CBCT.

We performed a leave-one-out cross validation to estimate the model’s stability and accuracy, which revealed very low variation in each model coefficient. So, the model is not strongly influenced by individual patients, showing that, even if the number of patients is low, the considered population is homogeneous enough for the model to reach stability. No outliers were extracted. These results are proof of the model’s good stability and accuracy.

The nomogram’s sensitivity was only 80 %, which is insufficient for clinical decision making. The nomogram failed to predict PG overdose for two patients, whom exhibited moderate mean PG dose increases (<1.5Gy). In order to improve the sensitivity and identify other anatomical parameters correlated with PG overdose, a larger patient cohort is required. In addition, an external cohort is needed to validate the nomogram before it can be implemented in routine clinical practice.

We attempted to translate the dosimetric benefit from ART to a clinical benefit, based on a xerostomia NTCP model. A strong relation existed between the mean doses to the parotid glands and salivary flow, and the efficacy of these NTCP models proved relatively high (area under the curve: 0.68–0.75) [24, 32–34]. Non-dosimetric patient factors, such as age, tumor stage, baseline xerostomia, and chemotherapy [32, 35–37] may also increase the risk of xerostomia, though this is controversial [32–34]. Using IMRT, severe xerostomia was detected in approximately 40 % of the patients, compared to 80 % using 3D conformal radiotherapy [38, 39]. The dose constraints recently suggested by the QUANTEC group [34] may enable reducing severe xerostomia to under 20 % [33, 37]. However, these dose constraints were only met in a minority of patients. In our study, the estimated xerostomia risk for the cumulated dose without replanning was 37 %, close to the observed xerostomia values in IMRT studies [38, 39]. For the patients predicted to receive overdose by the nomogram (70 % of our population), an ART strategy could enable the xerostomia risk to be reduced to under 30 %.

The key issues affecting ART use include the choice of image registration method to monitor the cumulated dose and thus trigger replanning, within a dose-guided radiotherapy perspective. The PGs are often close to or within a high-dose gradient, and even minor geometrical registration errors can lead to high cumulated-dose errors. A recent study evaluating accuracy for dose accumulation of 10 deformable-image registration methods in HNC [40]. For the most accurate method (FFD with mutual information), a mean cumulated dose point-to-point error of 2.5Gy for the PG was shown. Taking into account these uncertainties, it was possible to correctly identify the overall under- and overdosed PGs. Moreover, potential loss of PG cells during the course of radiotherapy [41] should be carefully considered due to the uncertainty of point-to-point matching between different CTs when using a deformable registration method. In addition, due to the possible heterogeneity of radiosensitivity within the PGs [42–44], local hotspots may also have different impacts on xerostomia risk. In our study, the predicted value of PG overdose was obtained from the estimated cumulated dose using the Dose Index Registry (DIR), and is therefore subject to uncertainty. For all these reasons, and in the interest of clinical justification (correlation between the mean PG dose and xerostomia), we only investigated the mean PG dose. Clearly, more thorough analysis into correlations between the PG cumulated dose and xerostomia, using a larger patient cohort or phantom-based studies evaluating the accuracy of the DIR, need to be carried out in order to validate the dose accumulation method for daily clinical practice.

## Conclusion

Based on the planning and first week CTs, our nomogram enabled the identification of all patients with large PG overdoses (≥2.5Gy). Replanning of these targeted patients lead to an eventual 4-Gy decrease in the mean PG overdose, while still respecting the dose-volume constraints in the other OARs and PTV. Other external cohorts are now required to validate this nomogram, as are clinical studies in order to validate the benefit of ART in the aims of decreasing the risk of xerostomia in locally-advanced HNC IMRT.

## Abbreviations

∆CTV_70_, CTV_70_ CT0-to-CT1 difference; ∆DosePG, difference between the mean PG doses of CT1 and CT0; ART, Adaptive Radiotherapy; CBCT, Cone-beam CT; CTV, Clinical Target Volume; FFD: free-form deformation; GTV, Gross Tumor Volume; LAHNC, locally-advanced head and neck carcinoma; NTCP, normal tissue complication probability; OAR, Organ at risk; PG, Parotid gland; PTV, Planning Target Volume.
